# Is amniotomy a risk factor for severe perineal trauma? -A Swedish nationwide register study

**DOI:** 10.1186/s12884-026-09367-9

**Published:** 2026-06-02

**Authors:** Sofia Tallhage, Kristofer Årestedt, Kristina Schildmeijer, Marie Oscarsson

**Affiliations:** 1 Faculty of Health and Life Sciences, Linnaeus University, Kalmar, 391 82 Sweden; 2 Department of Obstetrics and Gynecology, Region Kalmar County, Kalmar, 392 44 Sweden; 3 Department of Research, Region Kalmar County, Kalmar, 39244 Sweden

**Keywords:** Amniotomy, Severe perineal trauma, Labour interventions, Risk factors, Register study

## Abstract

**Background:**

Severe perineal trauma (SPT) is a serious complication of vaginal birth with potential physical and psychological consequences. Amniotomy is a commonly used labour intervention with uncertain evidence. A recent Norwegian study suggested a possible association between amniotomy and SPT. The aim of the study was to investigate whether amniotomy is independently associated with SPT, and whether the timing of amniotomy affects the occurrence of SPT in Swedish nulliparous and multiparous women.

**Methods:**

This nationwide register-based study included 403 342 women who gave birth between 2015 and 2020. Data were obtained from the Swedish Pregnancy Register. The main outcome, SPT, was defined using International Classification of Diseases (ICD-10) diagnosis codes O70.2 and O70.3. Associations were assessed using binary and multiple logistic regression analyses, with adjustment for maternal, obstetric, and neonatal factors.

**Results:**

The incidence of SPT was 5.1% among nulliparous and 0.9% among multiparous women. In unadjusted analyses, amniotomy was associated with increased odds of SPT in both nulliparous [OR 1.11; 95% CI, 1.07–1.16] and multiparous women [OR 1.13; 95% CI, 1.03–1.23]. However, these associations were not present in adjusted analyses [nulliparous: OR 1.00; 95% CI, 0.95–1.05; multiparous: OR 0.96; 95% CI, 0.86–1.07]. Early amniotomy was associated with a higher prevalence of obstetric interventions, and labour characteristics linked to SPT risk, consistent with confounding by indication. In adjusted analyses, timing of amniotomy was not associated with SPT in multiparous women, while in nulliparous women a longer interval between amniotomy and birth was associated with lower odds of SPT.

**Conclusions:**

Amniotomy was not independently associated with severe perineal trauma after adjustment for maternal, obstetric, and neonatal factors in this large nationwide cohort. The observed associations with timing likely reflect underlying labour complexity rather than a causal effect. These findings support a cautious and individualised use of amniotomy in clinical practice.

## Background

Amniotomy is a common intervention in obstetric and midwifery practice [1, 2]. Amniotomy is performed for various reasons, e.g., to induce labour, to accelerate labour when labour is prolonged, to receive information about the quality of the amniotic fluid, to enable the use of intrauterine pressure catheter and when internal fetal monitoring devices with a scalp electrode are wanted [2–7]. However, the main indication for its usage is to speed up labour, and thereby shorten the length of labour [2]. While its accelerating effect on labour progression is a generally held belief by clinicians, the evidence to support amniotomy for this purpose is uncertain [2]. Amniotomy has a long tradition, but its popularity has varied [2, 3]. There is no clear evidence that the potential benefits outweigh the potential harms despite its everyday use in clinical practice [4]. The recognized complications are cord compression leading to fetal heart decelerations, ascending infection rate, bleeding from fetal or placenta vessels, and umbilical cord prolapse [2].

Severe perineal trauma (SPT) is the most severe form of perineal trauma, affecting 2.5–5.9% of women giving birth [4, 8–10]. These injuries occur more commonly in nulliparous compared to multiparous women [11, 12], and their impact may extend into both short-term and long-term consequences [13, 14]. The morbidity includes both physical and psychological effects such as anal and urinary incontinence, sexual dysfunction, and reduced quality of life [13–18]. The initial classification of perineal tears in Sweden is made by the midwife responsible for the birth, who also repairs first and second degree tears [19]. If SPT is suspected, it is confirmed and sutured by an obstetrician. Effective preventive practices for SPT are scarce, previous research shows the effect of having two midwives attending birth in the late second stage focusing on perineal protection, warm compresses, and massage of the perineum [20]. The etiology of SPT is multifaceted and previously recognized risk factors include Asian ethnicity, nulliparity, persistent occipital-posterior position, the prolonged second stage of labour, instrumental birth, and high birthweight [9, 15, 17, 21–23]. The authors in a recent Norwegian case-control study including 842 births of which 421 were cases and 421 controls, explored the risk factors for SPT and found amniotomy to be the strongest risk factor for both nulliparous and multiparous women. The Norwegian study concluded that attention to the indications for and the timing of amniotomy may be a hitherto unrecognized risk factor for SPT, however, the findings need to be replicated in larger multicenter or nationwide studies to investigate this in preventive strategies [24].

Greater knowledge of potential risk factors is crucial in order to improve the obstetric management for all women giving birth. The aim of the present study was thus twofold; (1) to investigate whether amniotomy is a risk factor for SPT, and (2) to examine whether the timing of amniotomy affects the occurrence of SPT in nulliparous and multiparous women giving birth in Sweden.

## Methods

### Design

This nationwide register study was based on data from the Swedish Pregnancy Register.

### Data sources

The Swedish Pregnancy Register is a nationwide quality register that aims to cover all births in Sweden. The register, which started in 2013, contains detailed information on pregnant women at antenatal, delivery, and postnatal care, as well as diagnoses and procedure codes for both mothers and infants according to the International Classification of Diseases 10th edition (ICD-10) system and its validity has been assessed as high [25, 26].

### Study participants

**Fig. 1:**
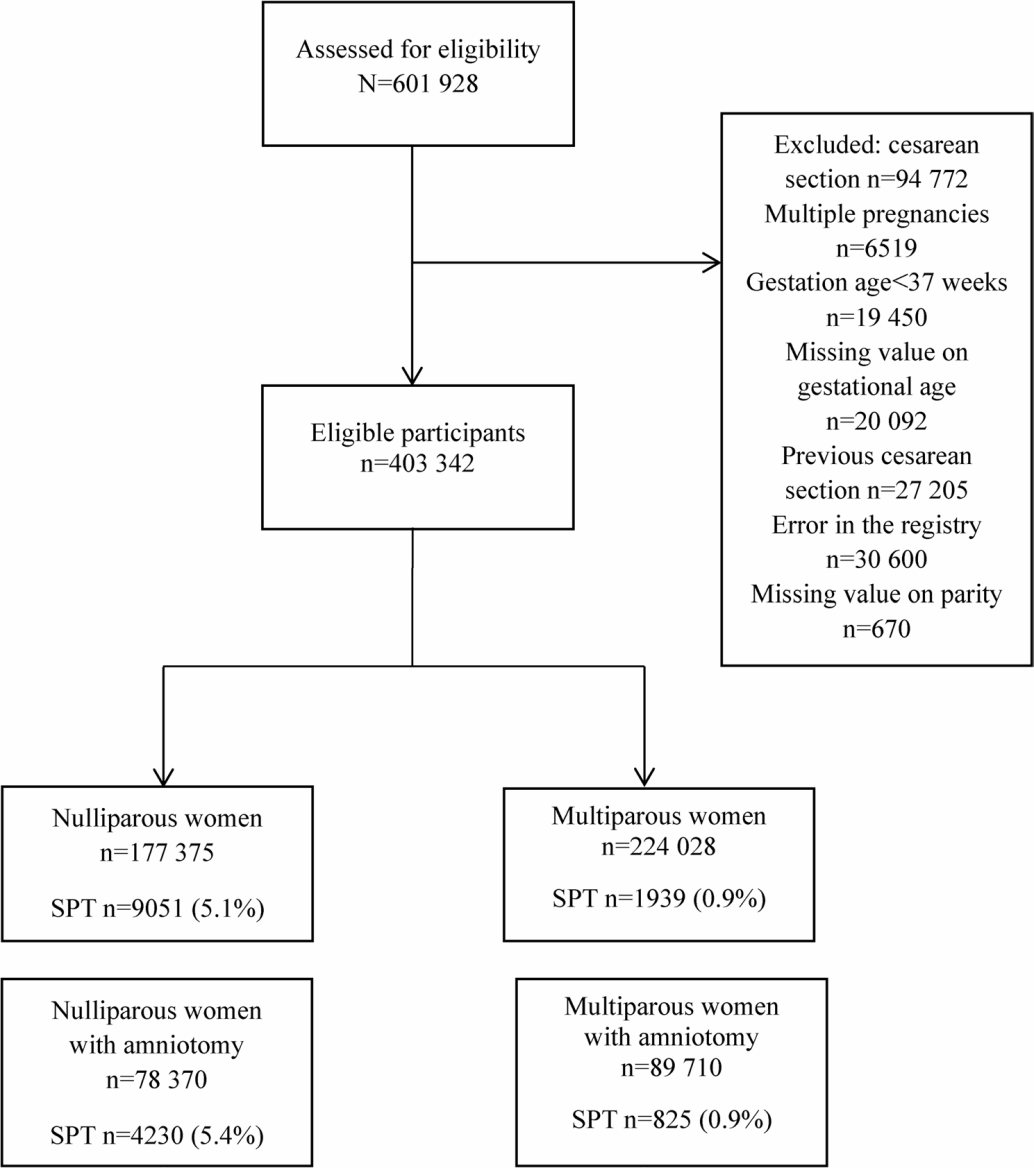
Flowchart of the study population

A total of 640 180 births occurred in Sweden between January 2015 to June 2020 of which 601 928 were registered in the Swedish Pregnancy Register. The register thus covered 94% of all births during the study period. Of the births, caesarean sections (*n* = 94 772), multiple pregnancies (*n* = 6519), gestational age < 37 weeks (*n* = 19 450), and missing values on gestational age (*n* = 20 092), were excluded. Women with a previous caesarean section have been shown to have an increased risk of obstetric anal sphincter injury in subsequent vaginal births [27]. To minimise confounding related to this elevated risk, women with a prior caesarean section (*n* = 27 205), were therefore excluded from the analysis. An error in the data set was identified during the data analysis as values on amniotomy and spontaneous rupture of the membranes were identical and inaccurate. The error was reported to the Pregnancy Register and confirmed; the error was caused by old versions of the medical record system being used. Subsequently, women with this error, *n* = 30 600, were excluded. A total of 670 of the births had missing data regarding parity and were therefore also excluded. The study population thus consisted of 403 342 births; 177 375 nulliparous, of whom 78 370 (42%) underwent amniotomy, and 224 028 multiparous women, of whom 89 710 (40%) underwent amniotomy (Fig. 1).

### Study variables

Maternal and neonatal variables were selected for the present study. The maternal variables included: SPT, maternal age, body mass index (BMI) recorded at the first antenatal visit, country of origin, onset of labour, amniotomy, spontaneous rupture of the membranes (SROM), use of epidural, augmentation with oxytocin, duration of active second stage of labour (pushing), episiotomy, instrumental delivery. The neonatal variables included gestational age, fetal presentation (categorised as occiput anterior, occiput posterior, breech, and other), birthweight, and head circumference.

SPT, i.e., third-degree and fourth-degree perineal tears involving the external sphincter muscle with or without an injury to the internal anal sphincter, anal epithelium, or rectum was identified in the data by the ICD-10 diagnosis codes O70.2 or O70.3 and the procedure code MBC33. The BMI was categorized according to the WHO standards into underweight (< 18.5), normal (18.5–24.9), overweight (25.0–29.9), and obesity (≥ 30.0). Maternal country of birth was categorized as born in Sweden, born outside of Sweden but in the EU, and born outside of the EU. Birthweight was categorized according to the WHO standards into low (< 2500 g), normal (2500–3999 g) and high (≥ 4000 g). Duration between amniotomy and birth was categorized into quartiles, different for nulliparous and multiparous women, as nulliparous women generally have longer labours.

### Statistical analysis

Descriptive statistics were used to present background variables. Comparisons between women with and without SPT were based on Student’s *t* test, Mann-Whitney U or Pearson chi-square (*χ*^*2*^) test according to variable type and distribution. Separate analyses were performed for the nulli- and multiparous women due to their differing baseline risk of severe perineal trauma.

Logistic regression analyses were used to explore amniotomy as a risk factor for SPT (aim 1). In a first step, univariate binary logistic regression analysis was used with SPT as outcome variable and amniotomy as explanatory variable. In a second step, multiple binary logistic regression analysis was used, including amniotomy as explanatory variable and already known and potential risk factors as adjusting covariates: maternal age, BMI, country of origin, use of epidural, augmentation with oxytocin, duration of active second stage of labour (pushing), episiotomy, instrumental delivery, fetal presentation (categorised as occiput anterior (reference), occiput posterior, breech, and other) and birthweight. Only birthweight was included in the regression model because birthweight, head circumference, and gestational age are highly intercorrelated, and including all three simultaneously would introduce redundancy and multicollinearity. Sensitivity analyses were performed restricting the sample to women with spontaneous onset of labour to assess robustness in a lower-risk population.

For aim 2, the analysis began with descriptive examinations of the timing of amniotomy (duration between amniotomy and birth according to quartiles) and its associations with obstetric characteristics and outcomes identified as significant risk factors for SPT. A longer amniotomy-to-birth interval corresponds to earlier amniotomy relative to birth, while a shorter interval corresponds to later amniotomy. Logistic regression analyses were then used to examine whether the timing of amniotomy affects the occurrence of SPT. Only the parturients who underwent amniotomy during labour were included in these analyses. In a first step, univariate binary logistic regression analysis was used with SPT as outcome variable and the duration between amniotomy and birth as explanatory variable. In a second step, multiple binary logistic regression analysis was used, including the duration between amniotomy and birth as explanatory variable and covariates of risk factors: maternal age, country of origin, use of epidural, augmentation with oxytocin, episiotomy, duration of active second stage of labour (pushing), instrumental delivery, fetal presentation (categorised as occiput anterior (reference), occiput posterior, breech, and other), and birthweight. Sensitivity analyses were performed within the subgroup of instrumental births, excluding duration of pushing and augmentation with oxytocin, as these variables may act as intermediates in the causal pathway between the explanatory variable and the outcome.

The results are presented with odds ratios (OR) and corresponding 95% confidence intervals (CI). The Hosmer-Lemeshow test was non-significant for all the multiple binary logistic regression models, which supports that the data fits the models.

A *p*-value of less than 0.05 was used to indicate statistical significance. The analyses were carried out using Stata IC 16.0 (StataCorp LLC, College Station, TX, USA).

## Results

The incidence of SPT was 5.1% in the total of 177 375 eligible nulliparous participants. The corresponding figure for the 224 028 eligible multiparous women was 0.9%. The mean age for nulliparous women was 29.0 (SD = 4.8) years, and the mean BMI was 24.4 (SD = 4.5). A total of 76% were born in Sweden and 15% outside of the EU. Induction of labour was used in 12%. A total of 86% had a spontaneous vaginal birth and 12% had an instrumental birth. The mean age for multiparous women was 32.0 (SD = 4.8) years, and the mean BMI was 25.2 (SD = 4.8). A total of 70% of the multiparous women were born in Sweden and 22% outside of the EU. Induction of labour was used in 10%. The majority (97%) had a spontaneous vaginal birth and 1.4% instrumental birth. Maternal, obstetric, and neonatal characteristics are presented for nulli- and multiparous women, with and without SPT, in Table 1.

**Table 1:** Maternal, obstetric and neonatal characteristics of the included nulliparous and multiparous women with and without severe perineal trauma (SPT)

	Nulliparous women (*n* = 177 375)		*p*-value	Multiparous women (*n* = 224 028)		*p*-value
	**SPT** (*n* = 9051) mean (SD) or *n* (%)	**Control** (*n* = 168 324) mean (SD) or **n (%)**		**SPT** (*n* = 1939) mean (SD) or **n (%)**	**Control** (*n* = 222 089) mean (SD) or **n (%)**	
Maternal age (years), mean (SD)	29.7 (4.6)	28.9 (4.8)	< 0.001^a^	32.7 (4.5)	32.0 (4.8)	< 0.001^a^
BMI (kg/m^2^), mean (SD)	24.4 (4.4)	24.3 (4.5)	0.507^a^	25.4 (4.7)	25.1 (4.8)	0.028^a^
Country of origin, n (%)			< 0.001^b^			< 0.001^b^
Sweden	5628 (68.1)	117 464 (76.3)		1156 (65.2)	142 367 (69.3)	
Born outside of Sweden but in the EU	673 (8.2)	13 200 (8.6)		124 (7.0)	17 678 (8.6)	
Born outside of the EU	1960 (23.7)	23 282 (15.1)		493 (27.8)	45 473 (22.1)	
Unkonwn	790	14 378		166	18 510	
Gestational age (weeks), mean (SD)	40.0 (1.2)	39.7 (1.2)	< 0.001^a^	39.8 (1.2)	39.5 (1.2)	< 0.001^a^
Induction of labour, n (%)	1201 (13.3)	19 894 (11.8)	< 0.001^b^	195 (10.1)	21 811 (9.7)	0.635^b^
Amniotomy, n (%)	4230 (46.7)	74 140 (44.0)	< 0.001^b^	825 (42.5)	88 885 (39.7)	0.010^b^
Epidural, n (%)	5919 (65.4)	97 713 (58.0)	< 0.001^b^	640 (33.0)	51 367 (22.9)	< 0.001^b^
Augmentation with oxytocin, n (%)	6351 (70.2)	98 265 (58.4)	< 0.001^b^	561 (28.9)	46 285 (20.7)	< 0.001^b^
Duration of pushing (hours), mean (SD)	0.76 (0.51)	0.66 (0.45)	< 0.001^a^	0.41 (0.38)	0.26 (0.25)	< 0.001^a^
Episiotomy, n (%)	1156 (12.8)	16 974 (10.0)	< 0.001^b^	87 (4.5)	3107 (1.4)	< 0.001^b^
Instrumental delivery, n (%)						
Vacuum extraction	2577 (28.5)	18 976 (11.3)	< 0.001^b^ regexm(moderns_diagnoser_i_en_rad, “O811”)	146 (7.5)	3100 (1.4)	< 0.001^b^
Forceps	30 (0.3)	144 (0.1)	< 0.001^b^	4 (0.2)	16 (0.0)	< 0.001^b^
Fetal presentation, n (%)			< 0.001^b^			< 0.001^b^
Occiput anterior	8417 (93.7)	159 686 (95.6)		1736 (90.8)	211 245 (95.3)	
Occiput posterior	454 (5.0)	5003 (3.0)		147 (7.7)	8358 (3.8)	
Breech	23 (0.3)	1475 (0.9)		5 (0.3)	836 (0.4)	
Other	91 (1.0)	916 (0.5)		23 (1.2)	1113 (0.5)	
Unknown	66	1244		28	2476	
Birthweight (gram), mean (SD)	3676 (466)	3483 (450)	< 0.001^a^	3895 (506)	3640 (470)	< 0.001^a^
Head circumference (cm), mean (SD)	35.4 (1.7)	34.9 (1.7)	< 0.001^a^	35.7 (1.7)	35.2 (1.6)	< 0.001^a^

SPT was significantly more common among women who underwent amniotomy compared to women who did not, in both nulliparous (5.4% vs. 4.9%, *p* < 0.001) and multiparous women (0.9% vs. 0.8%, *p* = 0.004). Amniotomy increased the odds for SPT in the simple binary regression analysis for both nulliparous- [OR 1.11; 95% CI, 1.07–1.16], and multiparous women [OR 1.13; 95% CI, 1.03–1.23]. The crude association between amniotomy and SPT disappeared after adjustment for maternal, obstetric, and neonatal factors for nulliparous women [OR 1.00; 95% CI, 0.95–1.05] and for multiparous women [OR 0.96, 95% CI, 0.86–1.07] (Table 2). Sensitivity analyses restricted to women with spontaneous onset of labour showed results consistent with the main analyses for both nulliparous and multiparous women.

**Table 2:** Multiple binary logistic regression analysis for severe perineal trauma

	Nulliparous, *n* = 177 375		*p*-value	Multiparous, *n* = 224 028		*p*-value
	OR	95% CI for OR		OR	95% CI for OR	
Amniotomy						
No	Reference			Reference		
Yes	1.00	0.95–1.05	0.957	0.96	0.86–1.07	0.417
Maternal age (years)	1.03	1.03–1.04	< 0.001	1.03	1.02–1.04	< 0.001
BMI (kg/m^2^) category						
< 18.5	1.33	1.17–1.52	< 0.001	1.04	0.71–1.51	0.857
18.5–24.9	Reference			Reference		
25-29.9	1.09	1.03–1.15	0.004	1.11	0.99–1.25	0.077
≥ 30	0.98	0.90–1.06	0.721	1.04	0.89–1.21	0.629
Country of origin						
Sweden	Reference			Reference		
Born outside of Sweden but in the EU	1.06	0.97–1.16	0.207	0.97	0.80–1.18	0.740
Born outside of the EU	1.97	1.86–2.09	< 0.001	1.56	1.38–1.76	< 0.001
Epidural						
No	Reference			Reference		
Yes	1.08	1.02–1.14	0.008	1.51	1.34–1.69	< 0.001
Augmentation with oxytocin						
No	Reference			Reference		
Yes	1.13	1.06–1.20	< 0.001	0.98	0.87–1.11	0.813
Duration of pushing (hours)	1.13	1.07–1.18	< 0.001	1.99	1.80–2.19	< 0.001
Episiotomy						
No	Reference			Reference		
Yes	0.78	0.72–0.85	< 0.001	2.00	1.53–2.62	< 0.001
Instrumental delivery						
No	Reference			Reference		
Vacuum extraction	2.71	2.54–2.88	< 0.001	2.52	1.97–3.23	< 0.001
Forceps	5.77	3.61–9.22	< 0.001	10.75	2.33–49.58	0.002
Presentation						
Occiput anterior	Reference			Reference		
Occiput posterior	1.50	1.33–1.69	< 0.001	1.69	1.38–2.06	< 0.001
Breech	1.54	0.98–2.44	0.063	1.36	0.50–3.67	0.545
Other	1.66	1.30–2.14	< 0.001	1.66	0.97–2.85	0.064
Birthweight (grams) category						
<2500	0.49	0.45–0.54	< 0.001	0.59	0.45–0.79	< 0.001
2500–3999	Reference			Reference		
≥4000	2.0	1.87–2.11	< 0.001	2.25	2.02–2.51	< 0.001

The risk factors that remained statistically significant in the multiple logistic regression for both nulliparous and multiparous women were increasing maternal age, origin outside of the EU, increasing duration of the active second stage of labour. Furthermore, epidural, instrumental delivery, occiput posterior presentation, and increasing birthweight, all increased the odds for SPT. Underweight according to BMI and the use of Oxytocin were identified as risk factors for the nulliparous women while episiotomy implied lower odds for SPT. Episiotomy instead increased the odds for SPT for multiparous women (Table 2).

The nulliparous women with amniotomy, *n* = 78 370, had a median amniotomy-to-birth interval of 5.7 h, compared with 1.7 h among multiparous women (*n* = 89 710). Women in the highest quartile of amniotomy-to-birth interval (i.e., earlier amniotomy relative to birth) had higher rates of epidural use, oxytocin augmentation, vacuum extraction, longer duration of pushing, higher mean birthweight, and more frequent occiput posterior presentation among nulliparous women (Table 3). Importantly, these factors were all independently associated with increased odds for SPT in the adjusted analyses, indicating that earlier amniotomy occurred in a higher-risk obstetric context. Similar associations were observed among multiparous women (Table 4), except for occiput posterior presentation. Women with longer duration between amniotomy and birth consistently exhibited a clustering of increased obstetric interventions and established risk factors for SPT. In the crude analyses, longer duration between amniotomy and birth was associated with increased odds of SPT in both nulliparous- [OR 1.04, 95% CI, 1.03–1.04], and multiparous women [OR 1.06, 95% CI, 1.04–1.08]. However, in the multiple logistic regression, increased duration was instead associated with decreased odds of SPT among nulliparous women, and no statistically significant association was observed among multiparous women (Table 5). Similarly, sensitivity analyses within the subgroup of instrumental births, excluding duration of pushing and oxytocin use, yielded results consistent with the main findings for both nulliparous and multiparous women.

**Table 3:** Associations of duration between amniotomy and birth, and risk factors for SPT in nulliparous women with amniotomy, *n* = 78 370

	< Q1	≥Q1, < Q2	≥Q2, < Q3	≥Q3	*p*-value
Epidural, *n* (%)	6708 (34.8)	12 938 (66.78)	16 103 (82.2)	17 894 (90.6)	< 0.001^a^
Augmentation with oxytocin, n (%)	5045 (26.2)	12 650 (65.3)	16 694 (85.2)	18 264 (92.4)	< 0.001^a^
Duration of pushing in hours, mean (SD)	0.56 (0.38)	0.65 (0.45)	0.68 (0.44)	0.77 (0.49	< 0.001^b^
Vacuum extraction, n (%)	1110 (5.8)	1803 (9.3)	2796 (14.3)	4889 (24.7)	< 0.001^a^
Occiput posterior, n (%)	412 (2.2)	609 (3.2)	738 (3.8)	792 (4.0)	< 0.001^a^
Birthweight in g, mean (SD)	3408 (432)	3499 (444)	3556 (452)	3660 (469)	< 0.001^b^

^a^Chi-Square test ^b^Anova < Q1 < 3.1 h, ≥Q1, < Q2 3.1–5.7 h, ≥Q2, < Q3 5.8–8.9 h, ≥Q3 > 8.9 h

**Table 4:** Associations of duration between amniotomy and birth, and risk factors for SPT in multiparous women with amniotomy, *n* = 89 710

	< Q1	≥Q1, < Q2	≥Q2, < Q3	≥Q3	*p*-value
Epidural, *n* (%)	2250 (10.1)	5180 (23.6)	8837 (39.6)	12 478 (55.2)	< 0.001
Augmentation with oxytocin, n (%)	578 (2.6)	2429 (11.1)	8977 (40.2)	16 912 (74.8)	< 0.001
Duration of pushing in hours, mean (SD)	0.19 (0.16)	0.28 (0.24)	0.30 (0.30)	0.34 (0.36)	< 0.001
Vacuum extraction, n (%)	165 (0.7)	275 (1.3)	374 (1.7)	776 (3.4)	< 0.001
Occiput posterior, n (%)	642 (2.9)	964 (4.4)	1102 (5.0)	1031 (4.6)	< 0.001
Birthweight in g, mean (SD)	3602 (450)	3676 (465)	3703 (486)	3746 (520)	< 0.001

^a^Chi-Square test ^b^Anova < Q1 < 0.6 h, ≥Q1, < Q2 0.6–1.7 h, ≥Q2, < Q3 1.8–3.8 h, ≥Q3 > 3.8 h

**Table 5:** Multiple binary logistic regression analysis for severe perineal trauma among women with amniotomy

	Nulliparous, *n* = 78 370		*p*-value	Multiparous, *n* = 89 710		*p*-value
	OR	95% CI for OR		OR	95% CI for OR	
Duration amniotomy-birth (hours) quartiles*						
< Q1	1.36	1.20–1.55	< 0.001	0.91	0.69–1.20	0.504
≥Q1, < Q2	1.12	1.01–1.25	0.026	0.95	0.74–1.21	0.660
≥Q2, < Q3	1.11	1.01–1.21	0.038	0.93	0.75–1.15	0.494
≥Q3	Reference			Reference		
Maternal age (years) category	1.03	1.02–1.04	< 0.001	1.03	1.01–1.05	< 0.001
Country of origin						
Sweden	Reference			Reference		
Born outside of Sweden but in the EU	1.09	0.95–1.24	0.194	0.94	0.69–1.28	0.685
Born outside of the EU	2.08	1.91–2.27	< 0.001	1.74	1.45–2.07	< 0.001
Epidural						
No	Reference			Reference		
Yes	1.15	1.05–1.26	0.002	1.44	1.21–1.70	< 0.001
Augmentation with oxytocin						
No	Reference			Reference		
Yes	1.32	1.20–1.45	< 0.001	0.91	0.75–1.11	0.353
Episiotomy						
No	Reference			Reference		
Yes	0.80	0.69–0.86	< 0.001	1.81	1.23–2.68	0.003
Duration of pushing (hours)	1.24	1.16–1.33	< 0.001	1.86	1.62–2.14	< 0.001
Instrumental delivery						
No	Reference			Reference		
Vacuum extraction	2.75	2.52-3.00	< 0.001	2.37	1.66–3.40	< 0.001
Forceps	4.93	2.54–9.57	< 0.001	14.89	1.55-142.81	0.019
Presentation						
Occiput anterior	Reference			Reference		
Occiput posterior	1.46	1.24–1.73	< 0.001	1.89	1.42–2.50	< 0.001
Breech	1.97	0.77–5.01	0.155	1.53	0.21–11.09	0.676
Other	1.45	0.99–2.12	0.056	2.75	1.44–5.26	0.002
Birthweight (grams) category						
< 2500	0.41	0.35–0.48	< 0.001	0.80	0.54–1.19	0.278
2500–3999	Reference			Reference		
≥4000	1.94	1.78–2.11	< 0.001	2.61	2.22–3.06	< 0.001

## Discussion

The present study aimed to investigate whether amniotomy is a risk factor for SPT, and to examine if the timing of amniotomy affects the occurrence of SPT, in nulliparous and multiparous women giving birth in Sweden. To the best of our knowledge, this is the largest study worldwide investigating the association between amniotomy and SPT. The study adds to the body of evidence on risk factors for SPT.

In this large population-based study, amniotomy was not independently associated with SPT after adjustment for maternal, obstetric, and neonatal factors. This differs from the findings in the Norwegian case-control study where amniotomy was identified as the strongest risk factor for SPT for both nulliparous and multiparous women [24]. The conflicting results are difficult to explain, however, the fact that the present study involves almost all births and hospitals in Sweden during the study period is a possible explanation. The Norwegian study included 421 women with SPT and 421 matched controls, compared to the present study including 11 138 women with SPT, and 406 743 women without SPT. The rates of amniotomy, regardless of parity and SPT, were higher in the present study compared to the Norwegian; in both nulliparous (43% vs. 15%) and multiparous women (39% vs. 15%), which also can contribute to the contradictory results.

Regarding the timing of amniotomy, earlier intervention (i.e., longer amniotomy-to-birth interval) was associated with a higher prevalence of obstetric interventions and established risk factors for SPT, indicating that it occurred in a more complex labour context. However, these associations are observational and likely reflect confounding by indication rather than causal effects. An amniotomy early in labour may be performed in response to slow labour progression, for example in the presence of a larger baby or epidural use [28], and prolonged labour is also an indication for instrumental delivery. While our findings do not support a causal effect of amniotomy on SPT, it is possible that amniotomy, as part of clinical management, may initiate a cascade of interventions in some cases [29]. This interpretation aligns with findings from our previous study [3], in which midwives described how amniotomy could trigger further interventions – a concern also reflected in other studies [29, 30].

In the adjusted analyses, the association between amniotomy-to-birth interval and SPT was inconsistent, with longer intervals (i.e., earlier amniotomy) associated with lower odds of SPT in nulliparous women and no significant association in multiparous women. These findings should be interpreted with caution, as they are likely influenced by residual confounding and reverse causation, where the timing of amniotomy reflects labour progression and clinical decision-making rather than an independent effect on SPT risk. One possible explanation for the observed pattern is that nulliparous women with shorter amniotomy-to-birth intervals (i.e., later amniotomy) may represent a group with more favourable or rapidly progressing labour, in whom amniotomy is performed at a more advanced stage. In such cases, amniotomy is likely to coincide with an already established labour trajectory. Strategies aimed at optimising the pace of birth and providing perineal support have been described in previous studies [8, 21, 31]. Within this context, our findings may underscore the importance of individualised clinical decision-making and suggest that amniotomy should be used only when clinically indicated [2–4, 32–34].

The adjusting covariates: increasing age, maternal origin outside of the EU, longer duration of active stage of labour, instrumental birth, occiput posterior presentation, and increasing birthweight, were identified as risk factors for SPT, which is in accordance with previous studies [9, 15, 17, 21–23, 35]. We also found that underweight according to the level of BMI was associated with an increased risk for SPT in nulliparous women, a result that is in line with observations reported in several earlier studies [12, 23, 35]. Epidural was also identified as a significant risk factor for SPT, in both nulliparas and multiparas. The opposite was found in a Danish population-based study including 214 256 nulliparous women, in which epidural was found as a significant protective factor for SPT [22]. The contradictory results of the effect of epidural analgesia on development of SPT may partly be explained by the different prevalence of the procedures, which vary considerably in the two studies. The rates of epidural in the nulliparous women in the present study vs. the Danish were (57.4% vs. 22.2%). This indicates different traditions of management of labour and birth, which may also influence the results. Several factors correlate with each other in clinical practice but based on our findings, epidural should be considered a risk factor of SPT.

The finding showed that SPT was more common in both nulliparous and multiparous women who had episiotomy performed during birth. Furthermore, episiotomy was associated with increased risk for SPT in multiparas in the adjusted analysis, while it was associated with decreased odds among nulliparas women. However, these findings should be interpreted with caution, as episiotomy is often performed in response to clinical situations associated with increased risk of SPT, such as instrumental delivery or suspected fetal compromise. This introduces a substantial risk of confounding by indication, making it difficult to determine whether the observed associations reflect a causal effect of episiotomy or the underlying risk profile.

The evidence concerning episiotomy preventing SPT is conflicting, while episiotomy may potentially be protective against SPT in some settings, others report it as a risk factor [8, 36–39]. However, studies show that a mediolateral episiotomy appears to have a protective effect for SPT in instrumental deliveries [9, 22, 40]. Sweden has in an international comparison a tradition of a low use of episiotomy, the current practice is restrictive and selective use, with episiotomy performed in 6.4% of spontaneous vaginal birth and in 32% of vacuum extraction, in nulliparous women [41]. According to the current Swedish standards, episiotomy in spontaneous birth should only be applied in the presence of non-reassuring fetal heart rate or shoulder dystocia [42]. The current recommendation for instrumental deliveries is that an episiotomy can reduce, but not eliminate, the risk of SPT. Episiotomy is an iatrogenic injury that can cause complications such as perineal pain, wound infection, and dehiscence. The importance of balancing the possible benefit with the possible consequences of an episiotomy itself, and to apply an individualised use based on clinical judgement has been argued [22, 39, 43].

A major strength of this study is the almost complete coverage of women giving birth in Sweden. The use of high-quality healthcare registers employing standardized records throughout the country and nonselective registration are major advantages regarding data ascertainment. There are some limitations with this study. First, there is limited evidence in relation to the risk of sustaining recurrent SPT in the subsequent birth [44]. The Swedish Pregnancy Register does not have any information on multiparous women who had SPT during a previous birth, which is why this could not be included in the analysis. Second, women with missing information on gestational age or parity were excluded from the analyses. This is a common occurrence in register-based studies and is unlikely to have substantially affected the overall findings, though it should be considered when interpreting the results. Third, very small differences appear significant in a large sample size as in the present study, even though they might be clinically irrelevant. Even if traditional effect size measures cannot be calculated for logistic regression models, odds ratio can serve as an effect size statistic. Fourth, the exposure definition based on quartiles of the amniotomy-to-birth interval may be difficult to interpret clinically, as these categories do not correspond to meaningful decision points in labour management. A classification based on labour stage or clinically defined early versus late amniotomy would have been preferable. However, such categorization was not possible, as the Swedish pregnancy register lacks information on cervical status at the time for amniotomy. Consequently, the timing variable may partly reflect labour progression rather than the effect of amniotomy itself. This is particularly relevant for the finding that shorter amniotomy-to-birth intervals were associated with higher odds of SPT among nulliparous women, which likely reflects reverse causation rather than a true causal relationship. These results should therefore be interpreted with caution. Future studies with detailed intrapartum data, including cervical dilation at amniotomy, are needed.

## Conclusions

Amniotomy was not independently associated with severe perineal trauma after adjustment for maternal, obstetric, and neonatal factors in this large nationwide cohort. The observed associations with timing likely reflect underlying labour complexity rather than a causal effect. These findings support a cautious and individualised use of amniotomy in clinical practice.

## Data Availability

The data that support the findings of this study are available from the Swedish Pregnancy Registry, but restrictions apply to the availability of these data, which were used under license for the current study, and so are not publicly available. Data are however available from the authors upon reasonable request and with permission of the Swedish Pregnancy Registry.

## Abbreviations

BMI: body mass index
CI: confidence interval
OR: odds ratio
SPT: severe perineal trauma
SROM: spontaneous rupture of the membranes
WHO: World Health Organization
